# Oncogenic role of epithelial cell transforming sequence 2 in lung adenocarcinoma cells

**DOI:** 10.3892/etm.2021.10220

**Published:** 2021-05-21

**Authors:** Hongyi Tan, Xiaoshan Wang, Xiaogang Yang, Haitao Li, Ben Liu, Pinhua Pan

Exp Ther Med 12: 2088-2094, 2016; DOI: 10.3892/etm.2016.3584

Following the publication of this paper, it was drawn to the Editors' attention by a concerned reader that certain of the cell Transwell assay data in the article (featured in Fig 4) were strikingly similar to data appearing in different form in another article by different authors, which was already under consideration for publication elsewhere at the time of the present article's submission.

Owing to the fact that the contentious data in the above article had been submitted for publication elsewhere in different form prior to its submission to Experimental and Therapeutic Medicine, the Editor has decided that this paper should be retracted from the Journal. The authors are in agreement that the article should be retracted. The Editor apologizes to the readership for any inconvenience caused.

